# Mineral trioxyde aggregate versus calcium hydroxide in apexification of non vital immature teeth: Study protocol for a randomized controlled trial

**DOI:** 10.1186/1745-6215-12-174

**Published:** 2011-07-13

**Authors:** Aurélie Beslot-Neveu, Eric Bonte, Bruno Baune, Raphaël Serreau, Fawzia Aissat, Laurent Quinquis, Sophie Grabar, Jean-Jacques Lasfargues

**Affiliations:** 1 Service d'Odontologie Hôpital Bretonneau, 2 rue Carpeaux 75018 Paris, France; 2 Centre de recherche clinique CIC-URC Necker Cochin Paris, URC Tarnier 89 rue d'Assas 75006 Paris, France; 3 Unité de Biostatistique et Epidémiologie, Hôpital Cochin, 27 rue du Faubourg Saint Jacques, 75014 Paris, France

## Abstract

**Background:**

Pulp necrosis is one of the main complications of dental trauma. When it happens on an immature tooth, pulp necrosis implies a lack of root maturation and apical closure. A therapy called apexification is required to induce the formation of a calcified apical barrier allowing a permanent and hermetic root filling. The aim of this prospective randomized clinical trial is to compare Mineral Trioxide Aggregate(MTA)with Calcium Hydroxide(CH)as materials used to induce root-end closure in necrotic permanent immature incisors.

**Methods/Design:**

This study, promoted by AP-HP, was approved by the ethics committee(CPP Paris Ile de France IV). 34 children aged from 6 to 18 years and presenting a non-vital permanent incisor are selected. Prior to treatment, an appropriate written consent has to be obtained from both parents and from children. Patients are then randomly assigned to either the MTA(experimental)or CH(control)groups. Recalls are performed after 3, 6 and 12 months to determine the presence or absence of a calcified apical barrier through the use of clinical and radiographic exams. Additional criteria such as clinical symptoms, apical radiolucencies, periapical index(PAI)are also noted.

**Trial registration:**

ClinicalTrials.gov no. NCT00472173 (First inclusion: May 10, 2007; Last inclusion: April 23, 2009; study completed: April 15, 2010)

## Background

Dental injuries are very common in children between six and nine years old. A serious complication of these traumas is the pulp necrosis whose prevalence varies with the type of traumatism from 1-6% for crown fractures to nearly 100% for intrusions [1]. The pulp necrosis of permanent immature teeth implies the interruption of the root formation and apical closure. It is then necessary to implement a therapy, called apexification to induce a hard calcific barrier at the apical end of the root, to achieve the definitive root canal filling. For a long time, calcium hydroxide was the only material used in the apexification procedure: The treatment consists in repeated stimulations with calcium hydroxide, over a six to eighteen months' period, until the apical closure is achieved [2]. Many studies in the literature report inconveniences linked to that procedure:

- The treatment involves numerous visits over a prolonged period and the patients may therefore fail to attend their appointments. It may also lead to a loss of temporary dressings and a re-infection of teeth. It is moreover impossible to perform a permanent restoration [3].

- In retrospective studies, cervical root fractures were said to occur on teeth during or following treatment with calcium hydroxide due to thin dentinal walls in immature teeth as well as to weakened dental structure induced by calcium hydroxide [4-7].

To avoid these inconveniences, the use of new materials potentially inducing mineralisation such as Mineral Trioxyde Aggregate(MTA)was suggested [8]. A review of the existing literature was done to determine if MTA could be considered an alternative to calcium hydroxide in apexification of permanent immature teeth. The analysis of in vitro and animal studies reveals different properties of MTA which seem interesting as regards apexification, offering good sealing ability [9-15], antimicrobial properties [11,16,17], a setting ability uninhibited by blood or water [11,18], biocompatibility, low cytotoxicity [19-22], non-resorbable nature [11,23] and also an effect on the induction of odontoblasts and of a hard barrier [11,24-30]. The MTA(Proroot^® ^MTA, Dentsply Maillefer France)is a medical device benefiting of EC marking. It is a powder mixed with sterile water to obtain a dental filling cement, used routinely for different clinical protocols in odontology. All clinical cases reported in the literature show, after a period of about six to twelve months, an absence of pathological clinical signs, the healing of a possible periapical lesion and the development of an apical closure [31-40]. However, the absence of a clinical comparative prospective study is to be deplored and we do not have the benefit of hindsight. That is why the implementation of a randomized clinical study in a public hospital is interesting to compare calcium hydroxide and MTA in the treatment of non vital immature teeth.

## Methods/Study design

### Objectives

- The main objective of this study is to test the ability of MTA, used as a root-end filling material, to induce a hard calcific barrier to seal hermetically non vital immature anterior permanent teeth with open apices over a 6 month's period.

MTA is a filling material allowing the creation of an artificial apical barrier-the MTA apical plug-but it is not good enough. The advantage of MTA is to induce a very close formation of mineralized tissue which is going to coat gradually the entire apex adhering to the MTA and the root walls. The interest of MTA is not only to create an immediate apical sealing(mechanical barrier)to induce a successful healing but also to promote the root-end growth or apexification, and finally the "bio-integration" of the non-vital immature permanent tooth.

- Secondary objectives:

• To assess the presence or absence of periapical radioluciency indicating the emergence or persistence of an apical periodontitis

• To compare the time required to obtain the clinical healing and the disappearance of clinical symptoms with the two materials: MTA and Calcium Hydroxide, when symptoms such as pain, swelling, sinus tract, abscess or abnormal mobility are present at the beginning of the study.

### Hypothesis

In children with pulp necrosis of a permanent immature incisor, MTA is not better than calcium hydroxide in the rates of a calcified apical barrier, but MTA is more valuable to achieve a biologic periapical barrier before 6 months.

### Research type

Randomized controlled trial(RCT), monocentric, open-labeled, superiority trial

### Subjects

The studied sample is composed of 34 children and teenagers, aged from 6 to 18 years presenting a pulp necrosis of a permanent immature incisor. The root maturation is normally completed 3 or 4 years after the tooth eruption, that is to say around the age of 9 or 10 years as far as incisors are concerned. However some teenagers present teeth damaged by a trauma before their maturation was achieved. That is the reason why such patients are included in the study when those traumas were not or unsuccessfully dealt with. The number of patients needed for this study was calculated prior to investigation with the hypothesis of a higher successful apexification rate in the MTA group compared to the control CH group. The anticipated success rates at 6 months were 5% in the CH group and 50% in the MTA group; Given these hypotheses, it was necessary to include 15 patients in each group that is to say a total of 30 patients to have a 80% statistical power(with a α risk of 5%), to compare the proportion of apical closure at 6 months with a Chi-2 test(two sided). Taking into account an anticipated dropout of 5%, it was decided to include 34 patients. Sample size calculations were computed with nQuery Advisor^® ^6.01. For ethical reasons, the sample size was limited while remaining compatible with the ability to obtain a significant difference between the two materials. The case reports published in the literature show a periapical repair within the first 6 months whereas the apical bridging induced with calcium hydroxide is more often obtained after a period of 6 to 18 months. That is why we hypothesized that after a period of 6 months, at least half of the MTA cases would be a success for the main objective as opposed to a minimal percentage for the calcium hydroxide group.

#### Inclusion criteria

I1-Patient presenting a non-vital permanent immature incisor for which an apexification treatment is indicated

I2-Aged 6-18 years

I3-Written informed consent obtained from each parent and from the child

I4-Medical exam

#### Exclusion criteria

E1-General pathology

E1.1-History of uncontrolled diabetes

E1.2-Immunosuppression

E1.3-Severe asthma

E1.4-Chronic systemic disease if a treatment is required

E1.5-Eating disorder(anorexia, bulimia, malnutrition, ...)

*E2-Oral pathology: *Periodontal disease

E3-Corticosteroid treatment during the last 3 months

E4-Non-affiliation to a social security scheme

#### Suspension criteria

S1-Poor compliance

S2-New trauma or new complication

S3-Intercurrent illness requiring discontinuation of the trial

When patients are lost sight of, observations must be noted down in the CRF until the last effected visit. The investigators will do their best to contact the patient and find out why the patient ceased being in the trial.

### Randomization

Participants are assigned in a treatment group by an independent statistician. Random numbers were randomly determined via a block randomization with a 1:1 ratio and blocks size of 4. The block size was concealed from all researchers. The randomization of the trial is centralized and conducted by an external authority: the Unity of Clinical Research(UCR). After the informed consent form is obtained, the investigator sends a randomization request fax to the UCR that sends back the random number according to the random sequence list. The latter fax is also sent to the hospital pharmacy that delivers the products necessary to the study.

### Design of the study-Interventions

For each patient the protocol is composed of 7 sessions(Table 1). The follow-up takes place over a 12 months' period. Day 0 is considered as the day of the first treatment session.

**Table 1:** Study protocol for each included patient

Sessions	1	2	3	4	5	6	7
**Timing**	D-15 to D0	D0	D + 15	D+21	3 months	6 months	12 months

**Written informed consent**	X						

**Inclusion and exclusion criteria**	X						

**Diagnosis of immature tooth pulp necrosis**	X						

**Clinical exam: symptoms**	X	X	X	X	X	X	X

**Radiographic exam**	X	X	X	X	X	X	X

**Calcium hydroxide filling or renewal**		X	X(CH group)		X(CH group-no barrier)	X(CH group-no barrier)	

**Apical filling with MTA**			X(MTA group)				

**Complete filling with gutta-percha**				X(MTA group)	X(CH group and apical barrier)	X(CH group and apical barrier)	

1. Day 0-14/Day 0: Inclusion and selection visit

Diagnosis of pulp necrosis, clinical and radiographic exams

Written informed consent is obtained from both parents and from the child.

2. Day 0: Calcium hydroxide canal conditioning regardless of the treatment group

This treatment is performed under local anesthesia with Articaïne with adrenalin 1/200000(Septanest, Septodont, France), after placement of a rubber dam. After preparing an access cavity and establishing the working length by taking a radiograph with a file inserted into the root canal within 1 mm of the radiographic apex, the canal is cleaned by irrigation with 3% sodium hypochlorite(Parcan, Septodont, France)and the use of manual files. The cleaning and shaping are realized with files with a very light parietal action to avoid the canal widening and the weakening of the root walls. Above all, it consists in removing the pulp remnants. The most important thing is to observe the vacuity and cleanliness of the canal using operative microscopy. Considering the root immaturity, it is actually a cleaning and desinfection without shaping. Then the canal is dried with paper point and can be filled with calcium hydroxide. Calcium hydroxide paste is prepared by mixing the calcium hydroxide powder(obtained from the hospital pharmacy)with an anaesthetic solution(Scandicaïne, Septodont, Saint Maur des Fossés, France). A plug of calcium hydroxide is deposed in the canal and condensed to the apical end of the root with a plugger. Other applications of calcium hydroxide are realized, until complete canal filling. The excess moisture is dried between each input with sterile paper points. The intracanal dressing quality is checked with a radiograph. The access cavity is temporarily sealed with a resin modified glass ionomer cement(Fuji II LC, GC, Bonneuil sur Marne, France). This calcium hydroxide canal conditioning is performed for all patients to allow the complete disinfection already obtained thanks to irrigation with sodium hypochlorite and the use of manual files, to control acute symptoms and to allow further treatments.

3. Day0+15: MTA apical filling for the testing group/calcium hydroxide renewal for the control group

This session starts with a local anesthesia, the placement of a rubber dam and the removal of all the calcium hydroxide. For this procedure, the canal has to be cleaned by irrigation and the use of ultrasonic files to remove all the calcium hydroxide. The use of EDTA allows a more complete elimination but due to these teeth immaturity the EDTA is not recommended. Then, the treatment differs among the treatment group:

For the MTA group, a MTA plug is placed into the canal with a root canal messing gun and condensed to the apical end of the root with a plugger to create a 4 mm apical plug of MTA. The pipe of the MTA gun is adjusted for the piston to deliver a 3 mm plug. Coarse paper points are used upside down to perfect the MTA adaptation to the apical walls. A radiographic control allows the practitioner to verify the good placement of MTA. A paper point moistened with sterile water is placed in the canal to produce an ambient humidity for the MTA to achieve its solidification. The access cavity is filled with conventional glass ionomer cement(Fuji IX, GC, Bonneuil sur Marne, France).

- For the CH group, the complete filling with calcium hydroxide is obtained as the first treatment session.

4. Day 0+21: Control for the CH group/complete filling with gutta-percha for the MTA group. The hardness of the MTA is checked with an endodontic file and the root canal filling with gutta-percha can be realized in contact with MTA. This filling is realized with the Schilder's technique: warm gutta-percha compacted with the Touch'n Heat(SybronEndo, Henry Schein, Alfortville, France). The access cavity is then sealed with a composite resin.

5. 3 months: clinical and radiographic control for all patients, and calcium hydroxide renewal for CH group patients.

6. 6 months: clinical and radiographic control for all patients and for CH group patients, calcium hydroxide renewal or complete root filling with gutta-percha if an apical barrier is present.

7. 12 months: clinical and radiographic control

For the teeth of CH group not filled with gutta-percha, the following treatment is realized outside study.

All the treatment sessions are realized in the same dental office, under local anesthesia, after placement of a rubber dam and with optical aids, by the same two operators, one assistant and the other alternately.

**Figure 1:**
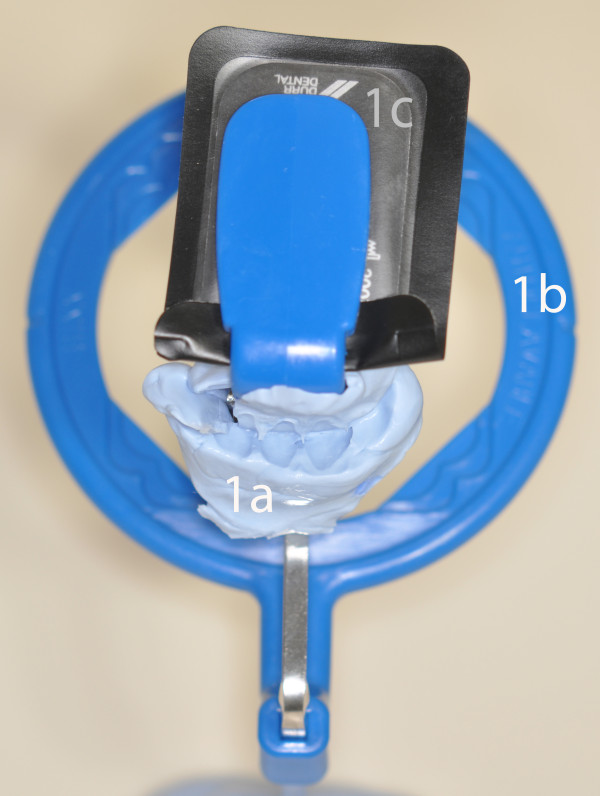
**Device used to realize reproducible radiographs at each session**. The patient is asked to bite into a piece of silicone, which enables us to put the film holder and so the radiograph in the same position at each session. 1a: piece of silicone. 1b: film holder. 1c: Radiograph.

**Figure 2:**
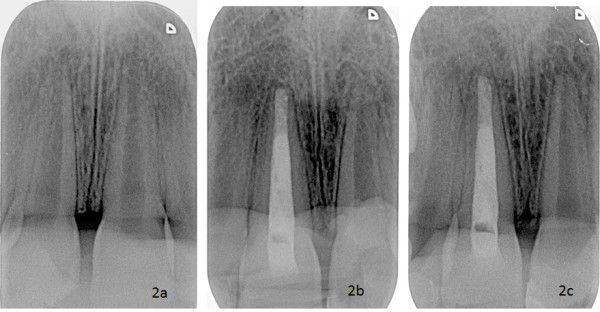
**The reproducibility of the radiographs taken at each session allowing us to compare them**. 2a: Radiograph taken during the inclusion session. 2b: Radiograph taken at the 6th month session. 2c: radiograph taken at the 12th month session.

The X-Rays of each patient are standardized so that they can be compared. When each patient has his first X-Ray taken, the patient is asked to bite into a piece of silicone which enables the operator to put the film holder in the same position at each session(Figure 1a, b, c). Thus the radiographs can be reproducible.(Figures 2a, b, c)

### Evaluation criteria

The assessment criteria are both clinical and radiographic(Table 2).

**Table 2:** Data reported in the CRF

Visits Data	V1	V2	V3	V4	V5	V6	V7
**Sex**	X						

**Age**	X						

**Clinical criteria**							

**Pain**	X	X	X	X	X	X	X

**Mobility(I, II, III, IV)**	X	X	X	X	X	X	X

**Swelling**	X	X	X	X	X	X	X

**Sinus tract**	X	X	X	X	X	X	X

**Abcess**	X	X	X	X	X	X	X

**Percussion tenderness**	X	X	X	X	X	X	X

**Radiographic criteria**							

**Degree of immaturity: Nolla stages: 7, 8, 9**	X						

**Endodontic filling**	X						

**Root resorption**	X						

**Periodontal ligament enlargement**	X	X	X	X	X	X	X

**Presence of an apical barrier**	X				X	X	X

**Radioluscent image**	X	X	X	X	X	X	X

**PAI**	X	X	X	X	X	X	X

**Form of the apical barrier**					X	X	X

#### Assessment with the patient at each session

First, the evaluation is realized at each session by the investigators-operators, who assess the presence or absence of clinical symptoms such as pain, abnormal mobility, swelling, sinus tract, abcess and percussion tenderness. Radiographic exams allow the evaluation of the main success criterion, the presence or absence of a calcified apical barrier as well as the presence of a radioluscent image and the form and thickness of the apical barrier. These investigators can compare all the patient's radiographs.

All these data are immediately entered in the CRF.

#### Anonymous evaluation at the end of the study

Two independent investigators will study all the anonymized X-Rays. This assessment will be done without the investigators having any information about the treated patients, their treatment or the nature of the × rays(preoperative, postoperative, after 3, 6 or 12 months). If the conclusions of the two investigators diverge, a third one will settle the matter. These anonymous data will be sent to the UCR that will then remove the anonymity before sending the service statistics for analysis.

### Expected benefits

The dental root canal can be hermetically filled immediately after the MTA hardens thus reducing the number of sessions and the length of the treatment.

### Predictable risks

Many *in Vitro *and *In Vivo *studies on MTA biocompatibility have been realized [21,25,26,41]. Ribeiro et al.(2006)demonstrated in an in vivo study, the absence of harmful effects of MTA [42]. Souza et al.(2006)carried out a study about the toxicity of 6 dental biomaterials and proved MTA was the least harmful of all [43]. This protocol does not involve any risks of serious side-effects entailed by the treatment.

### Undesirable events

Any adverse event observed during the study or the follow-up has to be reported in the CRF. Some information including occurrence time, duration, severity and treatment of this undesirable event should be noted as well. The relationship between adverse event and the apexification treatment has to be assessed(biological and mechanical failure).

### Monitoring and inspection

Monitor should regularly visit the dental office to control the process of study and the CRF, check storage of investigational dental products and record of data.

### Legal and ethical considerations

This study has been promoted by AP-HP(Paris Public Hospital)and approved by the ethics committee(CPP Paris Ile de France IV)and other French institutions:

- CNIL: French data protection watchdog

- AFSSAPS: the French Medecine and healthcare products regulatory agency, equivalent of the Food and Drug Administration in the United States.

Informed consent form(ICF)has been reviewed and approved by the ethics committee before study. Information about the protocol should be given to children and their parents both orally and in writing in an easily understandable language. The ICF must be dated and signed by the child and the parents(or representatives), and 3 copies must be made to be kept by the participants, the investigators and the UCR(15 years).

All information about the participants must be kept confidential.

### Statistical analysis

All the analyses followed an intention-to-treat principle and will be conducted to compare the efficacy of MTA with CH to induce root-end closure in necrotic permanent immature incisors at 6 months. Secondary efficacy endpoints are apical radiolucencies and PeriApical Index(PAI = 1, 2: healthy or PAI = 3, 4, 5: pathological)

Primary endpoints, ie, proportions of patients with an apical barrier close at 6 months will be compared between treatment groups with the Fisher's exact test, such as with the secondary endpoints.

To handle missing data on the primary endpoint, several procedures will be used where the missing values will be either dropped-out or replaced according to 3 simple imputation methods as follows:

- Observed data: patients with missing values are dropped out.

- Worst-case analysis: missing values are considered as a failure(apical barrier not closed at 6 months)in MTA group and as a success in CH group.

- Best-case analysis: missing values are considered as a success(apical barrier closed at 6 months)in MTA group and as a failure in CH group.

- Last Observation Carried Forward(LOCF):previous diagnostic is used to replace missing values.

The assessment of concordance between the two independent investigators about the closure of apical barrier at baseline, months 3, months 6 and months 12 will be given with the Kappa coefficient and the 95% confidence interval.

Analyses will be performed by two statisticians of Biostatistics and Epidemiology unit of Cochin Hospital(Paris)using SAS 9.1(SAS Institute Inc, Cary, North Carolina); tests will be 2-sided, and 0.05 will be used as the threshold for statistical significance.

## List of abbreviations

AFSSAPS: Agence Française de Sécurité Sanitaire des aliments et produits de santé; AP-HP: Assistance Publique-Hôpitaux de Paris; CH: Calcium Hydroxide; CNIL: Commission Nationale Informatique et Liberté; CRF: Case Report Form; EDTA: Ethylenediaminetetraacetic acid; ICF: Informed Consent Form; MTA: Mineral Trioxide Aggregate; PAI: Periapical Index; UCR: Unity of Clinical Research

## Competing interests

The authors declare that they have no competing interests.

## Authors' contributions

ABN, RS, JJL: participated in the design of the study. RS: is in charge of the trial management. FA is in charge of monitoring. BB manage the dental materials. ABN, JJL: do treatment and data collection. ABN, EB, JJL: are analyzing X-rays data. LQ, SG: are responsible of the statistical analysis. ABN particularly developed this publication. JJL: chair.

All authors read and approved the final manuscript.

## Author details

Aurélie Beslot-Neveu: DDS, Hôpital Bretonneau, Assistance Publique-Hôpitaux de Paris(AP-HP), Department of Pediatric Dentistry, Paris Descartes University, Unité de Recherche Biomatériaux Innovants et Interfaces EA 4462.

Eric Bonte: DDS, Associate Professor, Paris Descartes University, AP-HP, Hôpital Bretonneau, Department of conservative dentistry and endodontics, Paris

Bruno Baune: Hospital Pharmacist, AP-HP Hôpital Bretonneau, Paris

Raphaël Serreau: MD, PhD, pharmacologist, Project Manager, Unity of Clinical Research CIC-URC Necker Cochin Paris

Fawzia Aissat: Monitor, Unity of Clinical Research CIC-URC Necker Cochin Paris

Laurent Quinquis: Statistician, Unité de Biostatistique et d'Epidémiologie Hôpital Cochin, Paris

Sophie Grabar: MD, PhD, Paris Descartes University, AP-HP, Hôpital Cochin, Unité de Biostatistique et d'Epidémiologie, Paris

Jean-Jacques Lasfargues: DDS, Pr PhD, PD, Paris Descartes University, AP-HP, Hopital Bretonneau, Department of conservative dentistry and endodontics, Paris
